# Supplementary material for: Closed-loop control of gamma oscillations in the amygdala demonstrates their role in spatial memory consolidation
Source: Nat Commun. 2019 Sep 3;10:3970. doi: 10.1038/s41467-019-11938-8 (PMC6722067; doi:10.1038/s41467-019-11938-8)
Supplement: Supplementary file 1 — Supplementary Information [file 41467_2019_11938_MOESM1_ESM.pdf]

Closed-loop optogenetic control of gamma oscillations

Demonstrates their role in cognitive processes

Kanta et al., 2019

Supplementary Information

Supplementary Figure 1:

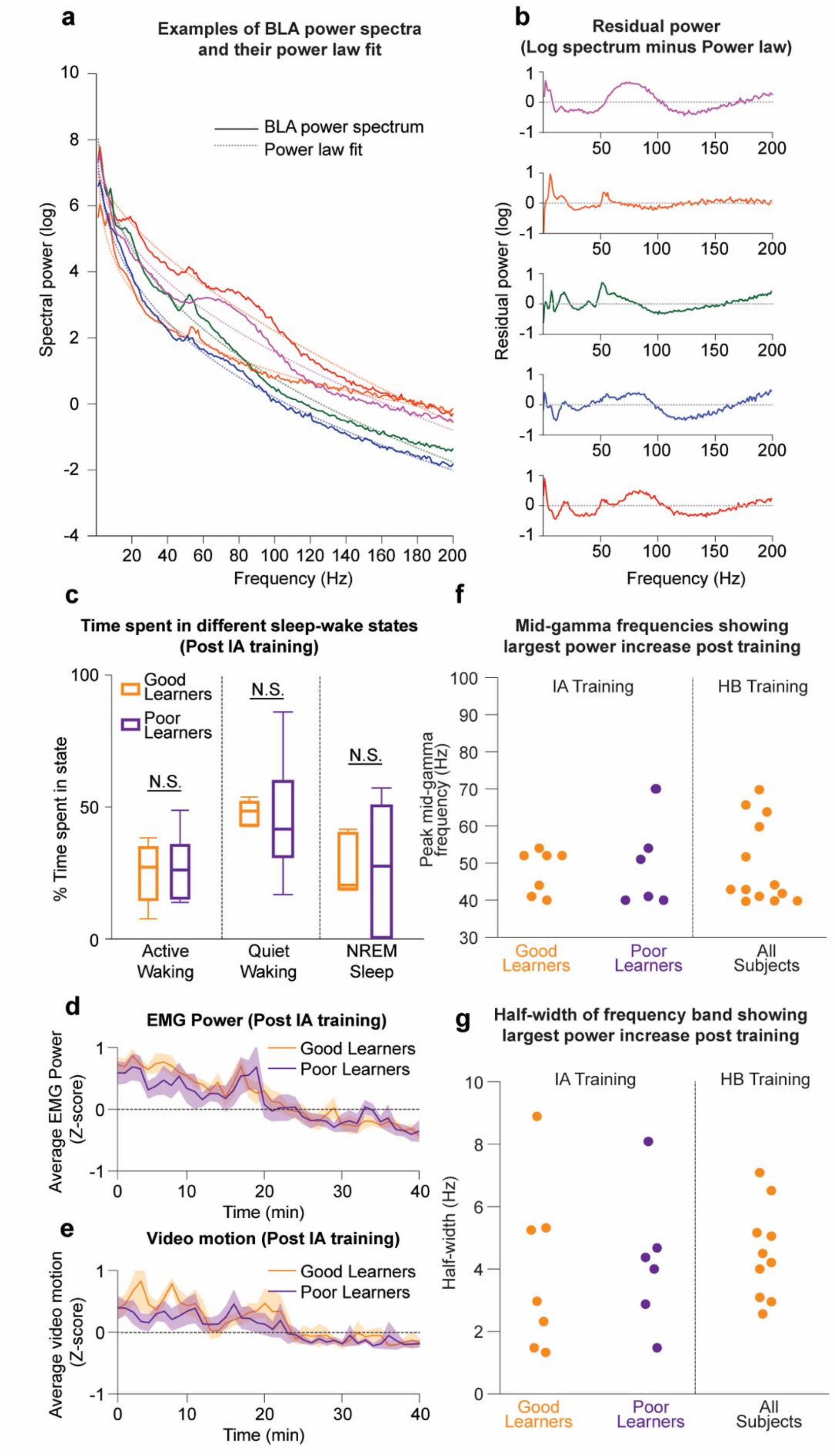

Supplementary Figure 1. Mid-gamma changes in BLA are not related to sleep-wake states and are similar across tasks. a, Plots of spectral power (log, solid lines) and their least squares power law regression (faint dashed lines). Each line pair represents one subject. **b**, Residual power is calculated by subtracting the power law from the raw spectrum. The purple, orange and green spectra are examples from IA subjects, the blue and red are from HB subjects. **c**, Percent time spent in Active Waking (AW, left), Quiet Waking (QW, center) and NREM sleep (NREM, right) for Good Learners (orange) and Poor Learners (purple; Good Learners: n=5 rats, Poor Learners: n=5 rats; AW, Mann Whitney U(9)=28, p=1; QW, Mann Whitney U(9)=32, p=0.42; NREM, Mann Whitney U(9)=27, p=0.94). **d**, EMG power after IA training in both groups. **e**, Average video motion after IA training in both groups. **f**, The mid-gamma frequency showing maximum power change post-training for IA (left, Good Learners: orange, n=7 rats, Poor Learners: purple, n=6 rats) and HB (right, All Subjects: orange, n=13 rats). Horizontal jitter for visualization purposes (Kruskal Wallis ANOVA $\chi^2(2)=0.22$, p=0.896). **g**, Half-width of frequency band that shows the largest power increase after the IA and HB tasks, for all groups.

Supplementary Figure 2:

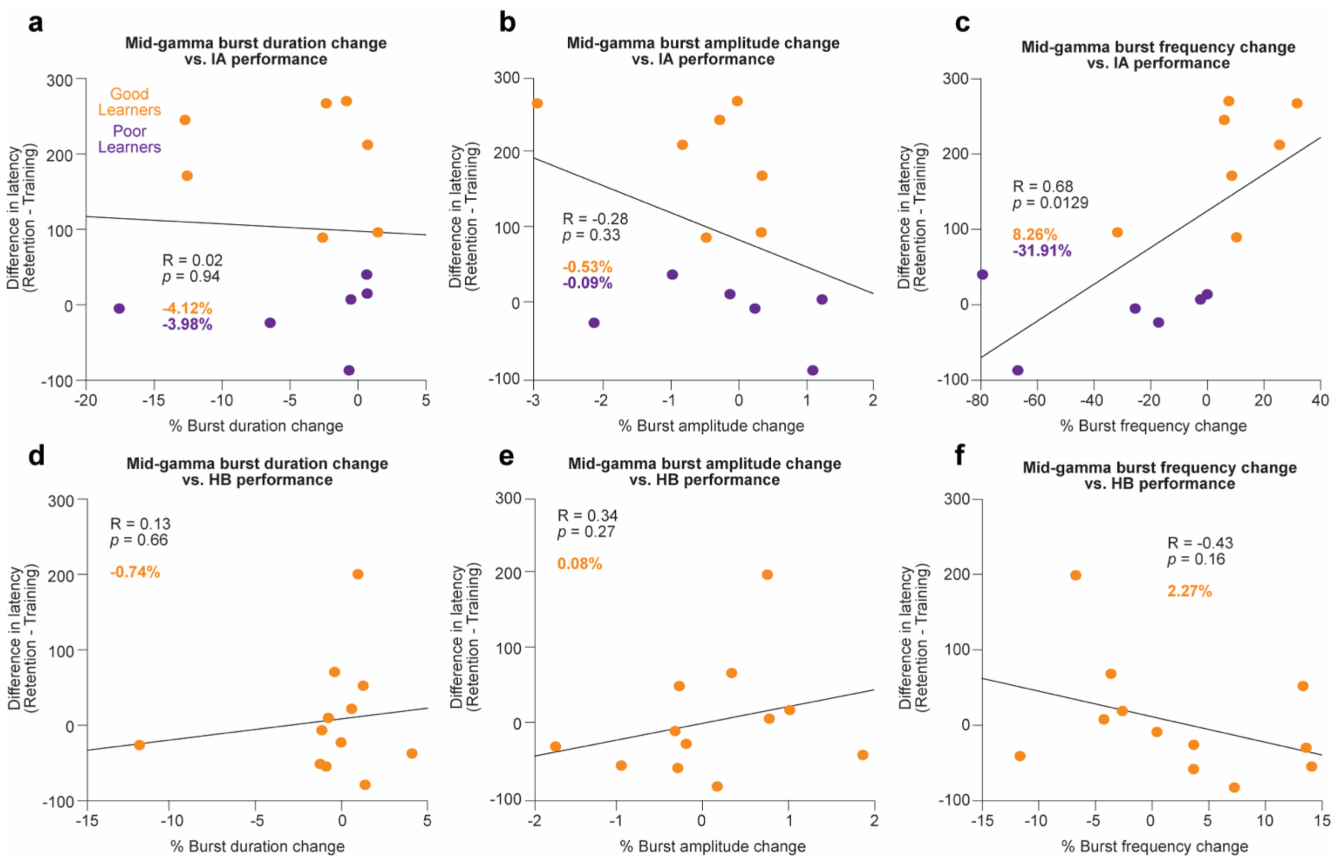

Supplementary Figure 2. Changes at the individual burst level caused by IA and HB training. **a**, Relationship between % change in individual burst duration and performance in IA (Spearman's rank-order correlation: R = 0.02, p=0.94). **b,** Relationship between % change in individual burst amplitude and performance in IA (Spearman's rank-order correlation: R = -0.28, p=0.33). **c,** Relationship between % change in individual burst frequency of occurrence and performance in IA (Spearman's rank-order correlation: R = 0.68, p=0.0129). **d,** Relationship between % change in individual burst duration and performance in HB (Spearman's rank-order correlation: R = 0.13, p=0.66). **e,** Relationship between % change in individual burst amplitude and performance in HB (Spearman's rank-order correlation: R = 0.34, p=0.27). **f,** Relationship between % change in individual burst frequency of occurrence and performance in HB (Spearman's rank-order correlation: R = -0.43, p=0.16).

Supplementary Figure 3:

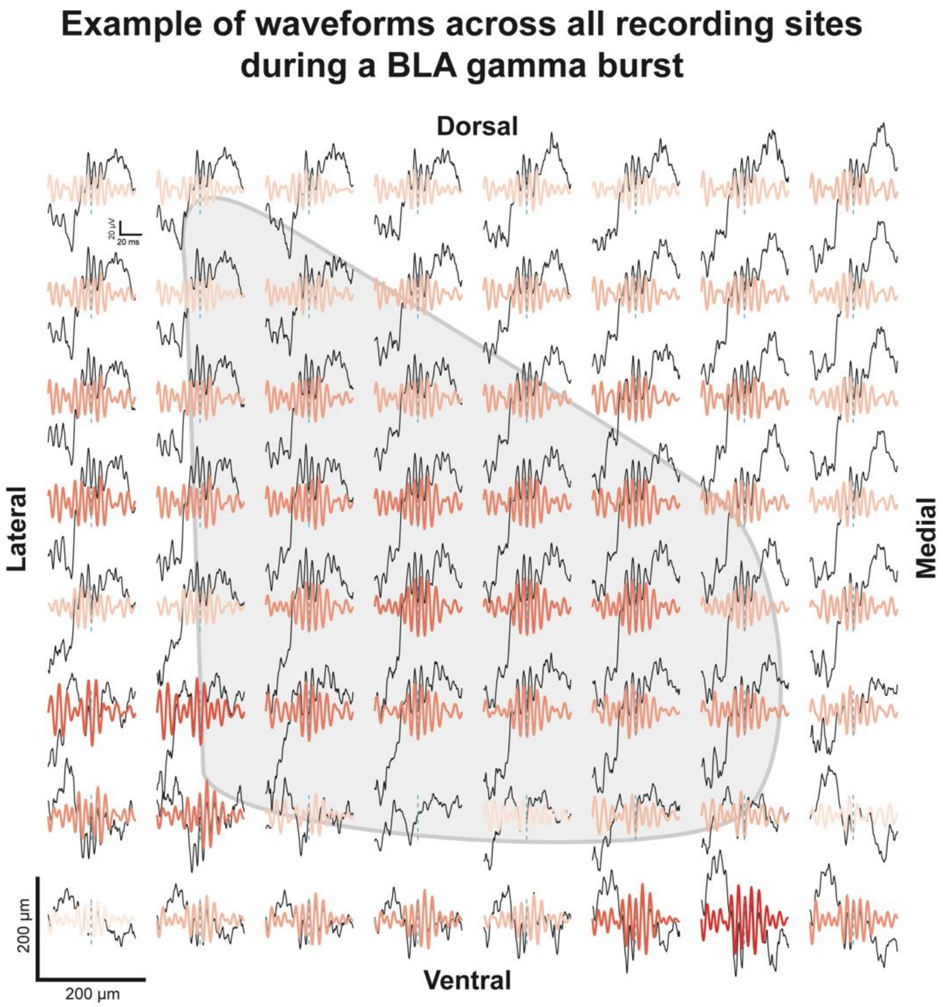

Supplementary Figure 3. Gamma bursts are strong and synchronous throughout the BLA. Example of a single gamma burst occurring in the BLA and simultaneous activity throughout all recording sites. Black traces: Raw LFP; Red traces: Filtered LFP. Blue dashed lines: First trigger in the burst. Grey shaded region: BLA.

Supplementary Figure 4:

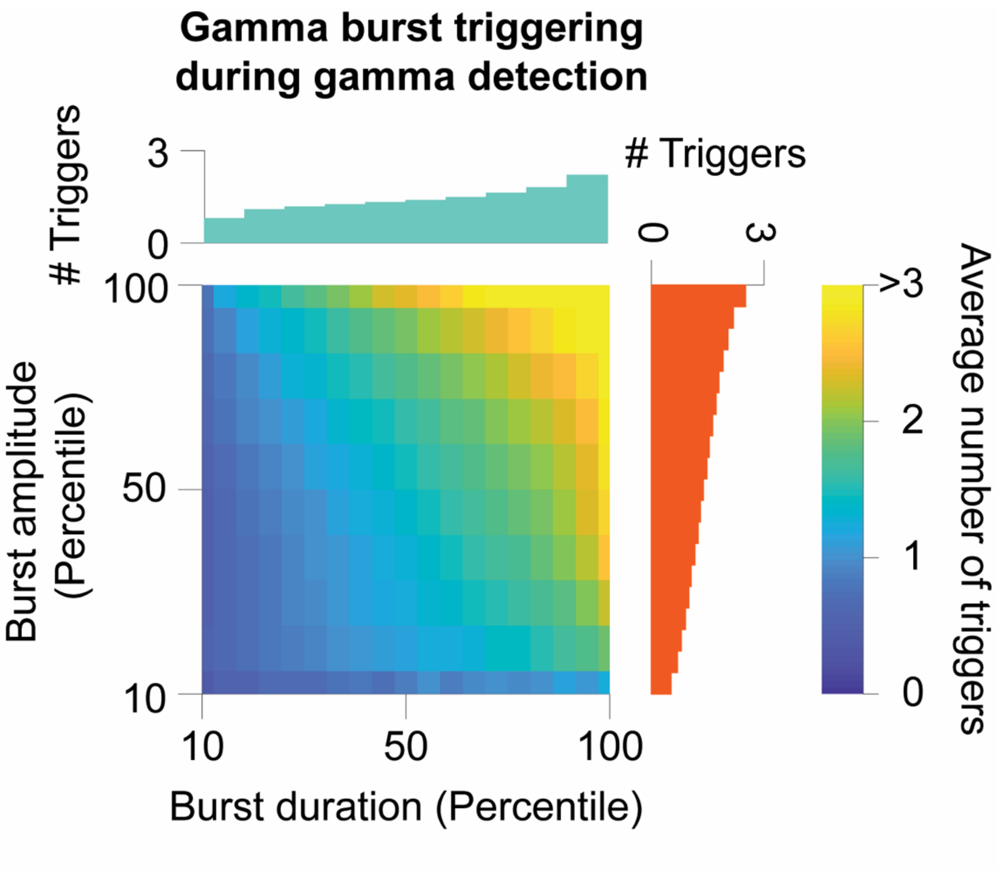

Supplementary Figure 4. Average number of triggers during mid-gamma bursts of different durations and amplitudes. Duration (x-axis) and amplitude (y-axis) is stratified by percentile value. Colors indicate number of triggers (color bar). Side bar graphs show marginal trigger counts.

Supplementary Figure 5:

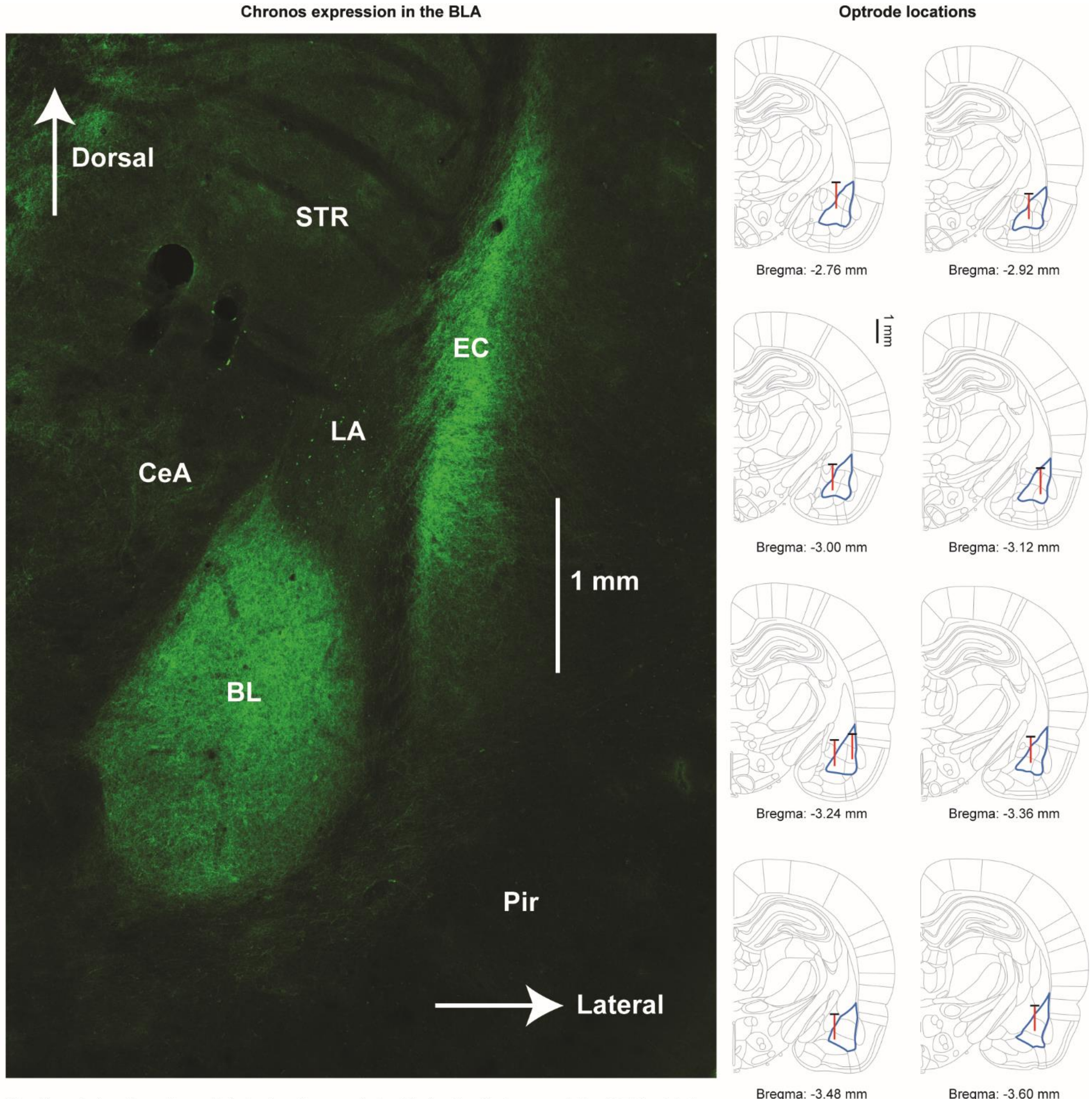

BL: Basolateral nucleus, LA: Lateral amygdala, CeA: Central amygdala, STR: striatum
EC: External capsule, Pir: Piriform cortex

Supplementary Figure 5. Typical opsin expression and locations of recording electrodes and optic fiber terminals for all gamma modulation animals. On the left, Chronos expression localized in the BLA. On the right, electrode locations. Electrodes, red; fiber tips, horizontal black lines above the electrodes. BLA is outlined in blue.

Supplementary Figure 6:

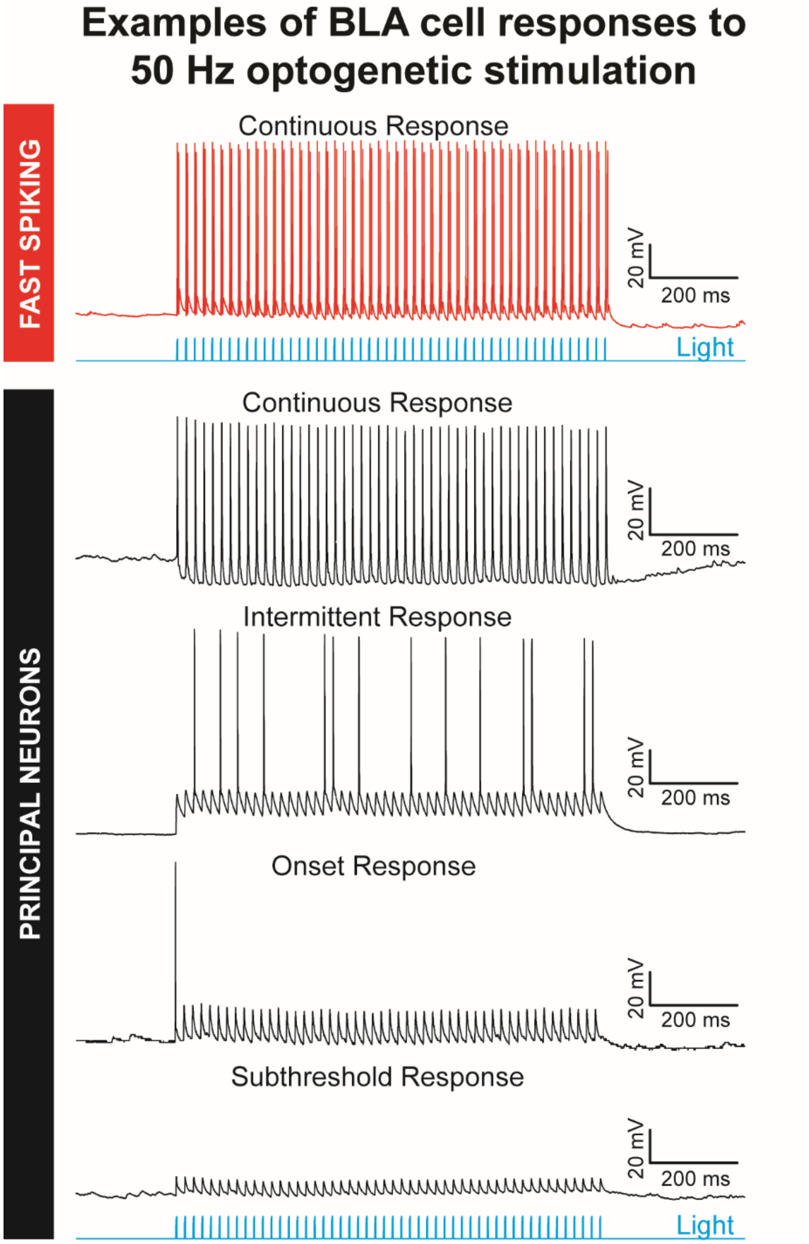

Supplementary Figure 6. Optogenetic responses of BLA PNs and FSs to 50 Hz light pulse trains. Only cells that produced action potentials in response to one of the other blue light stimulation protocols (e.g. 500 ms pulse) were used for this analysis. Example responses of FSs (top, red) and PNs (bottom, black) to the same 50 Hz light pulse train. Response types: Continuous Response, Intermittent Response, Onset Response, Subthreshold Response.

Supplementary Figure 7:

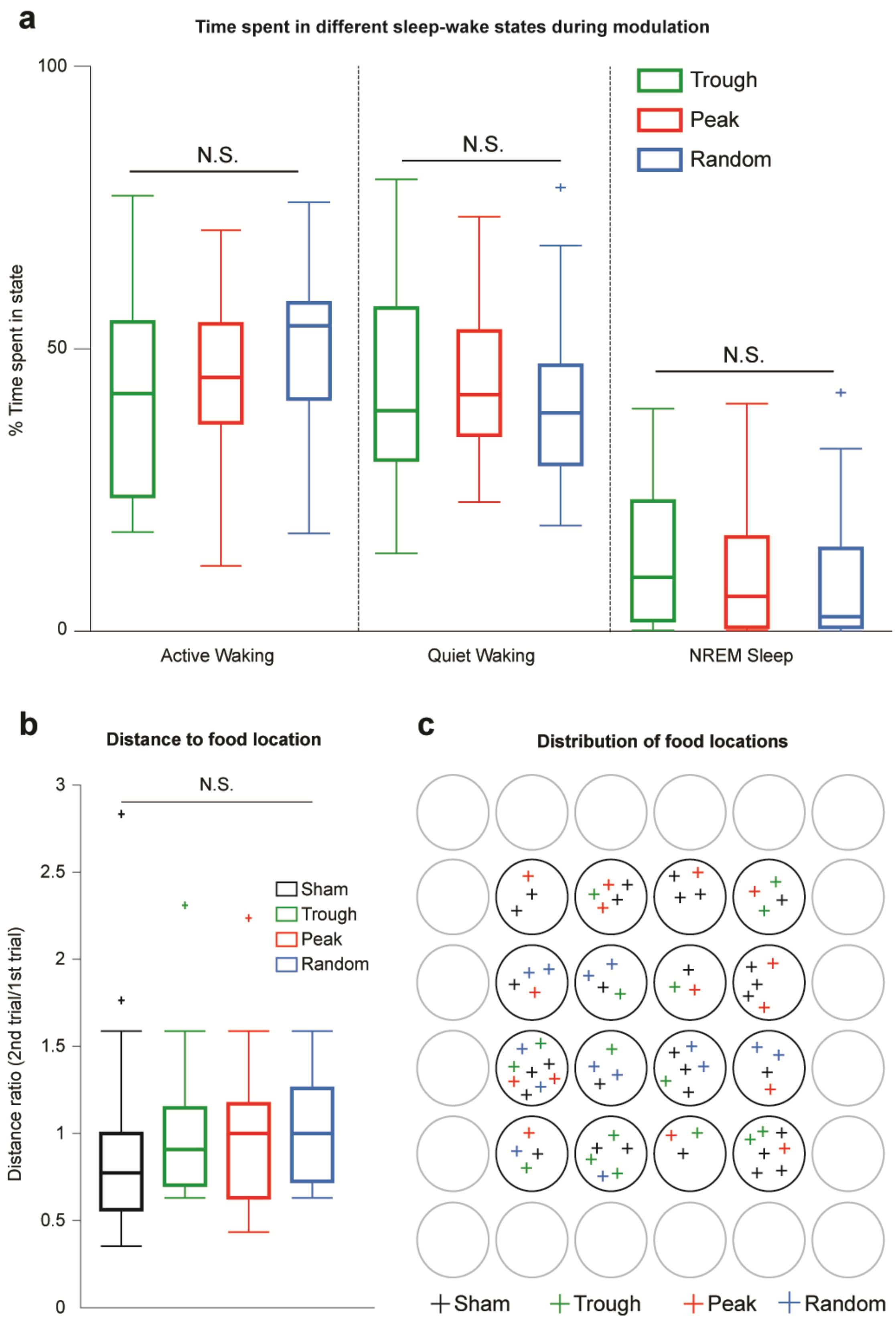

Supplementary Figure 7. Gamma levels and task performance changes are not due to sleep-wake state differences and are unrelated to task distances. a, Percent time spent in Active Waking (AW, left), Quiet Waking (QW, center) and NREM sleep (NREM, right); Trough-green (*n*=21 sessions, 8 rats), Peak-red (*n*=25 sessions, 9 rats), Random-blue (*n*=22 sessions, 9 rats) ; AW, Kruskal Wallis ANOVA $\chi^2(2)=4.19$, $p=0.123$; QW, Kruskal Wallis ANOVA $\chi^2(2) = 1.41$, $p = .493$; NREM, Kruskal Wallis ANOVA $\chi^2(2)=3.96$, $p=0.137$). **b**, Ratio of linear distances between entry point and food location (2nd trial distance/1st trial distance. Sham-black (*n*=30 configurations), Trough-green (*n*=16 configurations), Peak-red (*n*=15 configurations), Random-blue (*n*=14 configurations), Kruskal Wallis ANOVA $\chi^2(3)=3.24$, $p=0.35$).

c, Distribution of food locations in all treatment sessions (Sham-black, Trough-green, Peak-red, Random-blue). Each cross represents the food location on a different maze configuration.

Supplementary Table 1: List of animals and sessions used in HB experiments

**Subject**	**Session #**	**Notes**
B25	Sham: 5	*
	Trough: 2	
	Peak: 2	
	Random: 4 **(2)**	†
B26	Sham: 5	*
	Trough: 2	
	Peak: 2 **(1)**	†
	Random: 2	
B27	Sham: 2	‡
B28	Sham: 7	*
	Trough: 2 **(0)**	†
	Peak: 2	
	Random: 2	
B29	Sham: 6	*
	Trough: 2	
	Peak: 2	
	Random: 3	
B33	Sham: 3	‡
B36	Sham: 5	*
	Trough: 3 **(1)**	†
	Peak: 4 **(3)**	†
	Random: 2	
B43	Sham: 4	*
	Trough: 4 **(2)**	†
	Peak: 4	
	Random: 4 **(2)**	†
B46	Sham: 4	§
	Trough: 4	
	Peak: 3	
	Random: 4 **(2)**	†
B48	Sham: 4 **(2)**	†§
	Trough: 4	
	Peak: 4	
	Random: 4 **(3)**	†
	Sham: 4 **(3)**	†§
	Trough: 4	
	Peak: 4	
	Random: 4	
G3	Sham: 11	
G4	Sham: 6	‡

**Total *n***	**Fig. 1l-m**	49 sessions (subject avg. used in 1m)
	**Fig. 6b-e**<br>**Fig. 7a-f**	Sham: 30 sessions
		Trough: 21 sessions
		Peak: 25 sessions
		Random: 22 sessions
	**Fig. 7g-j**	Trough: 5 sessions
		Peak: 6 sessions
		Random: 6 sessions
	**Fig. 8**	Sham: 44 sessions (subject. avg used)
		Trough: 27 sessions (subject avg. used)
		Peak: 27 sessions (subject avg. used)
		Random: 29 sessions (subject avg. used)
